# Identification of a novel exon3 deletion of *RYR2* in a family with catecholaminergic polymorphic ventricular tachycardia

**DOI:** 10.1111/anec.12623

**Published:** 2019-01-07

**Authors:** Tommy Dharmawan, Tadashi Nakajima, Seiko Ohno, Takashi Iizuka, Shuntaro Tamura, Yoshiaki Kaneko, Minoru Horie, Masahiko Kurabayashi

**Affiliations:** ^1^ Department of Cardiovascular Medicine Gunma University Graduate School of Medicine Maebashi Japan; ^2^ Department of Bioscience and Genetics National Cerebral and Cardiovascular Center Suita Japan; ^3^ Department of Cardiovascular and Respiratory Medicine Shiga University of Medical Science Otsu Japan; ^4^ Center for Epidemiologic Research in Asia Shiga University of Medical Science Otsu Japan

**Keywords:** catecholaminergic polymorphic ventricular tachycardia, copy number variation, *KCNJ2*, *RYR2*, targeted panel sequencing

## Abstract

**Background:**

*RYR2*, encoding cardiac ryanodine receptor, is the major responsible gene for catecholaminergic polymorphic ventricular tachycardia (CPVT). Meanwhile, *KCNJ2*, encoding inward‐rectifier potassium channel (I_K1_), can be the responsible gene for atypical CPVT. We recently encountered a family with CPVT and sought to identify a responsible gene variant.

**Methods:**

A targeted panel sequencing (TPS) was employed in the proband. Copy number variation (CNV) in *RYR2* was identified by focusing on read numbers in the TPS and long‐range PCR. Cascade screening was conducted by a Sanger method and long‐range PCR. KCNJ2 wild‐type (WT) or an identified variant was expressed in COS‐1 cells, and whole‐cell currents (I_K1_) were recorded using patch‐clamp techniques.

**Results:**

A 40‐year‐old female experienced cardiopulmonary arrest while cycling. Her ECG showed sinus bradycardia with prominent U‐waves (≥0.2 mV). She had left ventricular hypertrabeculation at apex. Exercise induced frequent polymorphic ventricular arrhythmias. Her sister died suddenly at age 35 while bouldering. Her father and paternal aunt, with prominent U‐waves, received permanent pacemaker due to sinus node dysfunction. The initial TPS and cascade screening identified a *KCNJ2* E118D variant in all three symptomatic patients. However, after focusing on read numbers, we identified a novel exon3 deletion of *RYR2* (*RYR2*‐exon3 deletion) in all of them. Functional analysis revealed that KCNJ2 E118D generated I_K1_ indistinguishable from KCNJ2 WT, even in the presence of catecholaminergic stimulation.

**Conclusions:**

Focusing on the read numbers in the TPS enabled us to identify a novel CNV, *RYR2*‐exon3 deletion, which was associated with phenotypic features of this family.

## 1 INTRODUCTION

Catecholaminergic polymorphic ventricular tachycardia (CPVT) is characterized by exercise or emotion‐induced polymorphic or bidirectional ventricular arrhythmias (VAs) leading to syncope or sudden cardiac death in the absence of structural heart disease and is often accompanied by sinus node dysfunction (SND) or sinus bradycardia. (Leenhardt et al., 1995; Postma et al., 2005) (Miyata, Ohno, Itoh, & Horie, 2018) Several responsible genes for CPVT have been identified so far, and *RYR2* which encodes the cardiac ryanodine receptor (RyR2) accounts for approximately 60% of CPVT cases. (Laitinen et al., 2001; Priori et al., 2001).

The RyR2 is a calcium (Ca^2+^) release channel on the sarcoplasmic reticulum (SR) and plays a major role in the regulation of intracellular Ca^2+^ homeostasis. (Kushnir & Marks, 2010) In patients with *RYR2*‐related CPVT, intracellular Ca^2+^ overload due to an increased diastolic Ca^2+^ leakage from SR in the presence of high β‐adrenergic tone has been thought to be the cause of enhanced U‐waves on electrocardiogram (ECG) and VAs which may be coincident with delayed afterdepolarization (DAD)‐induced triggered activity. (Katra & Laurita, 2005; Katra, Oya, Hoeker, & Laurita, 2007; Lieve, van der Werf, & Wilde, 2016; Paavola et al., 2007; Viitasalo et al., 2008).

On the other hand, *KCNJ2*, encoding inward‐rectifier potassium current (I_K1_), has also been reported to be responsible for an atypical type of CPVT,(Tester et al., 2006) although *KCNJ2* is typically responsible for Andersen–Tawil syndrome (ATS) characterized by (a) VAs with prominent U‐waves (≥0.2 mV) on baseline ECG, (b) periodic paralysis, and (c) dysmorphic features. (Nguyen, Pieper, & Wilders, 2013; Plaster et al., 2001) However, approximately 30% of the disease‐causing *KCNJ2* variant carriers show only cardiac phenotypes. (Kimura et al., 2012).

We recently encountered a family with CPVT. Genetic screening of the proband by a targeted panel sequencing using next‐generation sequencer (NGS) identified only a rare *KCNJ2* E118D variant. However, after focusing on the read numbers of the NGS, we could identify a novel exon3 deletion of *RYR2* (*RYR2*‐exon3 deletion) without performing multiplex ligation‐dependent probe amplification (MLPA) method. To elucidate the causative roles of these variants, we investigated family members and functional role of the *KCNJ2* E118D variant.

## 2 METHODS

### 2.1 Clinical evaluation

**Figure 1:**
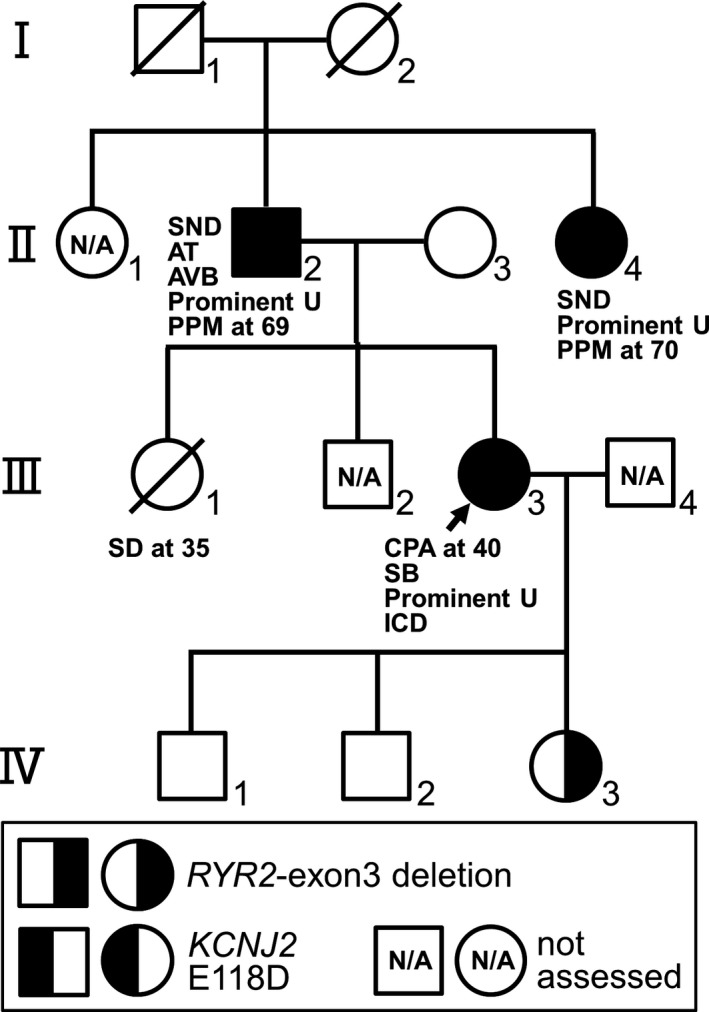
A pedigree and clinical characteristics of the family. AT, atrial tachycardia; AVB, atrioventricular block; CPA, cardiopulmonary arrest; ICD, implantable cardioverter defibrillator; PPM, permanent pacemaker; Prominent U, Prominent U‐waves at rest; SB, sinus bradycardia; SD, sudden death. Carriers of an exon3 deletion of *RYR2* (*RYR2*‐exon3 deletion) and a *KCNJ2* E118D variant are shown as the graphic symbols in the bottom

The proband (Ⅲ‐3) who had CPVT phenotype and her father (Ⅱ‐2), mother (Ⅱ‐3), paternal aunt (Ⅱ‐4), and three children (Ⅳ‐1, Ⅳ‐2, and Ⅳ‐3) were enrolled in this study (Figure 1). Baseline ECGs were available from all the subjects. Prominent U‐waves were defined as having U‐wave amplitude ≥0.2 mV on baseline ECG. Transthoracic echocardiograms (TTEs) were available from the proband (Ⅲ‐3) and her daughter (Ⅳ‐3). Hypertrabeculation or left ventricular non‐compaction (LVNC) localized at left ventricular apex (LVA) was defined as non‐compacted to compacted ratio of ≥2.0 when LVA was observed by short‐axis view of TTE as reported by Jenni et al.(Jenni, Oechslin, Schneider, Attenhofer Jost, & Kaufmann, 2001) The proband (Ⅲ‐3) and her daughter (Ⅳ‐3) underwent exercise stress test using ergometer (30‐watt ramp protocol). The proband (Ⅲ‐3) underwent isoproterenol infusion test (0.015 μg kg^–1^ min^–1^). The proband (Ⅲ‐3) also underwent left heart catheterization and subsequent acetylcholine provocation to coronary arteries (up to 100 μg), and programmed electrical stimulation up to three extra stimuli from both right ventricular apex and right ventricular outflow tract.

### 2.2 Genetic analysis

After obtaining appropriate approval from the institutional review board and written informed consent for the genetic analysis from all the subjects of this study, we performed genetic analysis.

Genomic DNA was extracted from peripheral blood lymphocytes as described previously.(Imai et al., 2014) We then performed a targeted panel sequencing of 55 genes of the proband (Ⅲ‐3) using NGS (MiSeq, Illumina, San Diego, CA, USA) (Table 1). Nucleotides substitution was confirmed by a Sanger method. To detect the copy number variations (CNVs), the depths (read numbers) of the NGS reads obtained by the targeted panel sequencing were compared between control and the proband by using the SureCall software (Agilent Technologies, Santa Clara, CA, USA). To confirm the CNV in *RYR2*, we performed long‐range PCR using KOD‐Plus‐Neo (Toyobo, Osaka, Japan). Forward primer (5′‐CACAGAACAGGACCAAGTTAGAGGC‐3′) and reverse primer (5′‐CATTACCTTCCTGACACACTTCATCCTAG‐3′) were designed to amplify a region that includes the deleted site of *RYR2*. The 5’ position of the forward primer and the reverse primer was 287,712 and 291,655 in the NCBI Reference Sequence NC_000001.10 (GRCh37), respectively. The PCR products were loaded into 0.8% agarose gels with Tris‐Acetate‐EDTA buffer and electrophoresed. Then, the bands were extracted and purified using the QIAquick Gel Extraction Kit (QIAGEN, Hilden, Germany), which were directly sequenced to confirm the precise location of the deletion in *RYR2*. Cascade screening of family members, her father (Ⅱ‐2), mother (Ⅱ‐3), paternal aunt (Ⅱ‐4), and three children (Ⅳ‐1, Ⅳ‐2 and Ⅳ‐3), was conducted by a Sanger method and a long‐range PCR.

**Table 1:** Fifty‐five genes analyzed by the targeted panel sequencing

AKAP9	ANKB	CACNA1C	CACNA2D1	CACNB2	CALM1
CALM2	CALM3	CAMK2D	CASQ2	CAV3	CTNNA3
DES	DPP6	DSC2	DSG2	DSP	GJA1
GJA5	GPD1L	HCN4	JUP	KCNA5	KCND3
KCNE1	KCNE2	KCNE3	KCNE4	KCNE5	KCNH2
KCNIP2	KCNJ2	KCNJ3	KCNJ5	KCNJ8	KCNN2
KCNQ1	LMNA	MOG1	NCS1	PKP2	RANGRF
RYR2	SCN10A	SCN1B	SCN2B	SCN3B	SCN4B
SCN5A	SLC8A1	SNTA1	TCAP	TMEM4	TRDN
TRPM4					

### 2.3 Mutagenesis and heterologous expression

Human KCNJ2 wild‐type (WT) cDNA (NM_000891.1) subcloned into pCMS‐EGFP vector (KCNJ2 WT) was used to engineer a KCNJ2 mutant.(Kimura et al., 2012) Site‐directed mutagenesis (KCNJ2 E118D) was performed by using the QuikChangeⅡSite‐Directed Mutagenesis Kit (Agilent Technologies, Santa Clara, CA, USA) according to the manufacturer's instructions. The presence of the mutation was confirmed by direct sequencing.

Using Lipofectamine 2000 (Invitrogen, Carlsbad, CA, USA), 1.0 μg of KCNJ2 WT cDNA or 1.0 μg of KCNJ2 E118D cDNA were transiently transfected into COS‐1 cells, and maintained in DMEM medium supplemented with 10% fetal bovine serum and 1% penicillin‐streptomycin in a 5% CO_2_ incubator at 37°C for 24–36 hr before current recordings. Cells exhibiting green fluorescence were chosen for the current recordings.

### 2.4 Electrophysiology

Membrane currents (I_K1_) were recorded using whole‐cell patch‐clamp techniques at room temperature (23–25℃). The bath solution for recording I_K1_ contained (in mmol/L) 140 NaCl, 5.4 KCl, 0.5 MgCl_2_, 1.8 CaCl_2_, 0.33 NaH_2_PO_4_, 5.5 glucose, 5 HEPES (pH 7.4 with NaOH), and the pipette solution contained (in mmol/L) 60 K‐aspartate, 65 KCl, 1 KH_2_PO_4_, 2 MgCl_2_, 3 EDTA, 3 ATP, 5 HEPES (pH 7.2 with KOH). The electrode resistance ranged from 1.0 to 2.5 MΩ when filled with pipette solution. Data acquisition was carried out using an Axopatch 200B amplifier and pCLAMP10.3 software (Molecular Devices, Sunnyvale, CA, USA). Currents were acquired at 20–50 kHz, and low pass‐filtered at 5 kHz using an analog‐to‐digital interface (Digidata 1440A acquisition system, Molecular Devices). Currents were evoked by 100 ms test pulses applied in 10 mV increments to potentials ranging from −140 mV to +50 mV from a holding potential of −60 mV. Current–voltage (I‐V) relationships were obtained by the current amplitudes at the end of 100 ms pulses against test potentials.

### 2.5 Catecholaminergic stimulation

To resemble the catecholaminergic effect (protein kinase A (PKA) activation) on KCNJ2 WT and KCNJ2 E118D, I_K1_ was also recorded 5 min after the presence of a PKA cocktail containing 100 μmol/L forskolin (Sigma‐Aldrich, Missouri, USA) and 10 μmol/L 3‐isobutyl‐1‐methylxanthine (Sigma‐Aldrich, Missouri, USA) in the Bath solution and 10 min after wash out of it as the same method reported by Vega et al.(Vega, Tester, Ackerman, & Makielski, 2009).

### 2.6 Statistical analysis

All data are expressed as mean ± *SE*, and statistical comparisons were tested using the unpaired Student's *t* test with *p* < 0.05 considered to be statistically significant.

## 3 RESULTS

### 3.1 Phenotypic manifestations

**Figure 2:**
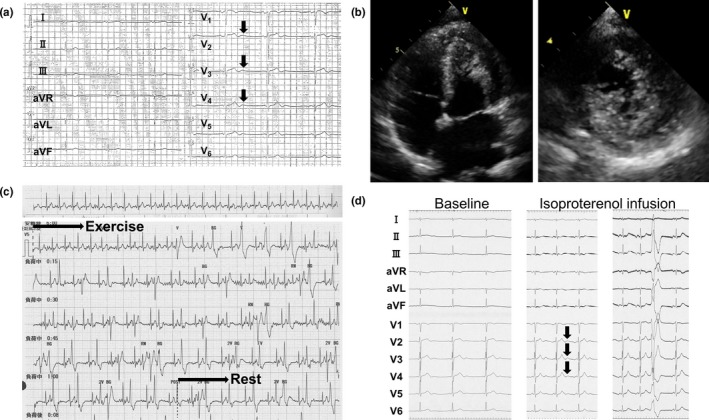
Clinical characteristics of the proband (Ⅲ‐3) (Figure 1). (a) 12‐lead ECG recorded at medical examination before cardiopulmonary arrest. Arrows indicate prominent U‐waves. (b) Four‐chamber view (left panel) and short‐axis view (right panel) during systole of transthoracic echocardiograms. Hypertrabeculation was recognized at left ventricular apex. (c) Exercise stress test using ergometer. D. Isoproterenol infusion test before (Baseline) and during isoproterenol infusion. Arrows indicate augmentation of U‐waves

**Figure 3:**
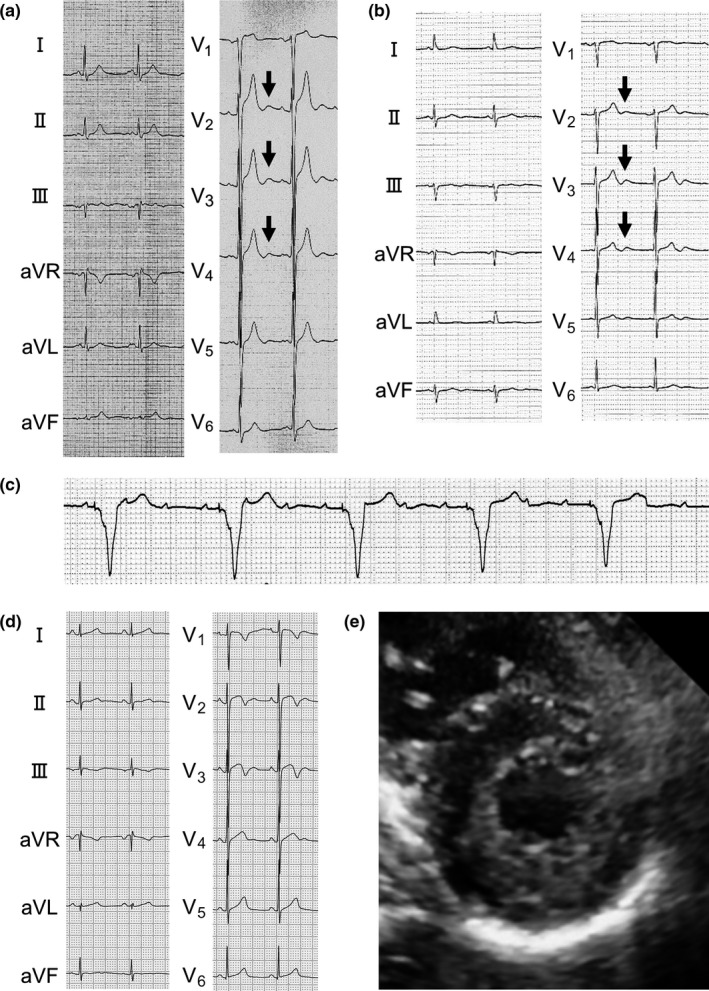
Clinical characteristics of the family (Figure 1). (a, b, and d) 12‐lead ECG of the proband's father (Ⅱ‐2) (a), paternal aunt (Ⅱ‐4) (b), and daughter (Ⅳ‐3) (d). Arrows indicate prominent U‐waves. (c) V_1_ lead of the proband's father (Ⅱ‐2) during ventricular pacing due to atrial tachycardia with atrioventricular block. (e) Short‐axis view during systole of transthoracic echocardiogram of the proband's daughter (Ⅳ‐3)

The proband (Ⅲ‐3) (Figure 1), a 40‐year‐old female, experienced cardiopulmonary arrest (CPA) while cycling and then was resuscitated. Her 12‐lead ECG recorded previously at medical examination showed sinus bradycardia (42 bpm) with prominent U‐waves (≥0.2 mV) in the anterior precordial leads (Figure 2a). Echocardiogram showed no structural heart diseases except for the presence of hypertrabeculation localized at left ventricular apex (Figure 2b) with preserved left ventricular systolic function (ejection fraction (EF): 73%). Exercise stress test induced frequent premature ventricular contractions (PVCs) and polymorphic VAs (Figure 2c). Notably, isoproterenol infusion test induced augmentation of U‐waves, followed by PVC (Figure 2d). Coronary angiography and subsequent acetylcholine provocation to coronary arteries revealed no significant stenosis in coronary arteries. Programmed right ventricular electrical stimulation did not induce VAs. Thus, the proband presented with typical phenotypic features of CPVT. An implantable cardioverter defibrillator (ICD) was implanted. However, it was extracted because of an ICD infection, then a subcutaneous ICD was implanted. Although medical therapies such as β‐blockers and flecainide were taken into consideration, those were not administered because of the sinus bradycardia phenotype. Under strict exercise restriction, a defibrillator shock has never been delivered. On family history, her elder sister (Ⅲ‐1) died suddenly while bouldering at the age of 35 (Figure 1). Her father (Ⅱ‐2) and paternal aunt (Ⅱ‐4), whose ECGs also showed prominent U‐waves at rest (Figures 3a,b), received permanent pacemaker (PPM) at the age of 69 and 70, respectively, due to SND (Figure 1). Her father (Ⅱ‐2) also had atrial tachycardia (AT) with atrioventricular block (AVB) (Figure 3c). Her three children (Ⅳ‐1, Ⅳ‐2, and Ⅳ‐3) had been asymptomatic. Her daughter (Ⅳ‐3)’s ECG showed normal sinus rhythm (72 bpm) with no prominent U‐waves but inverted T‐waves in V_1_–V_3_ leads (Figure 3d). Exercise stress test induced only one premature ventricular contraction. Her echocardiogram also showed hypertrabeculation localized at LVA (Figure 3e) with preserved systolic function (EF: 67%). Remaining family members in the pedigree have been asymptomatic.

### 3.2 Identification of a rare *KCNJ2* E118D variant and exon3 deletion of ***RYR2***

**Figure 4:**
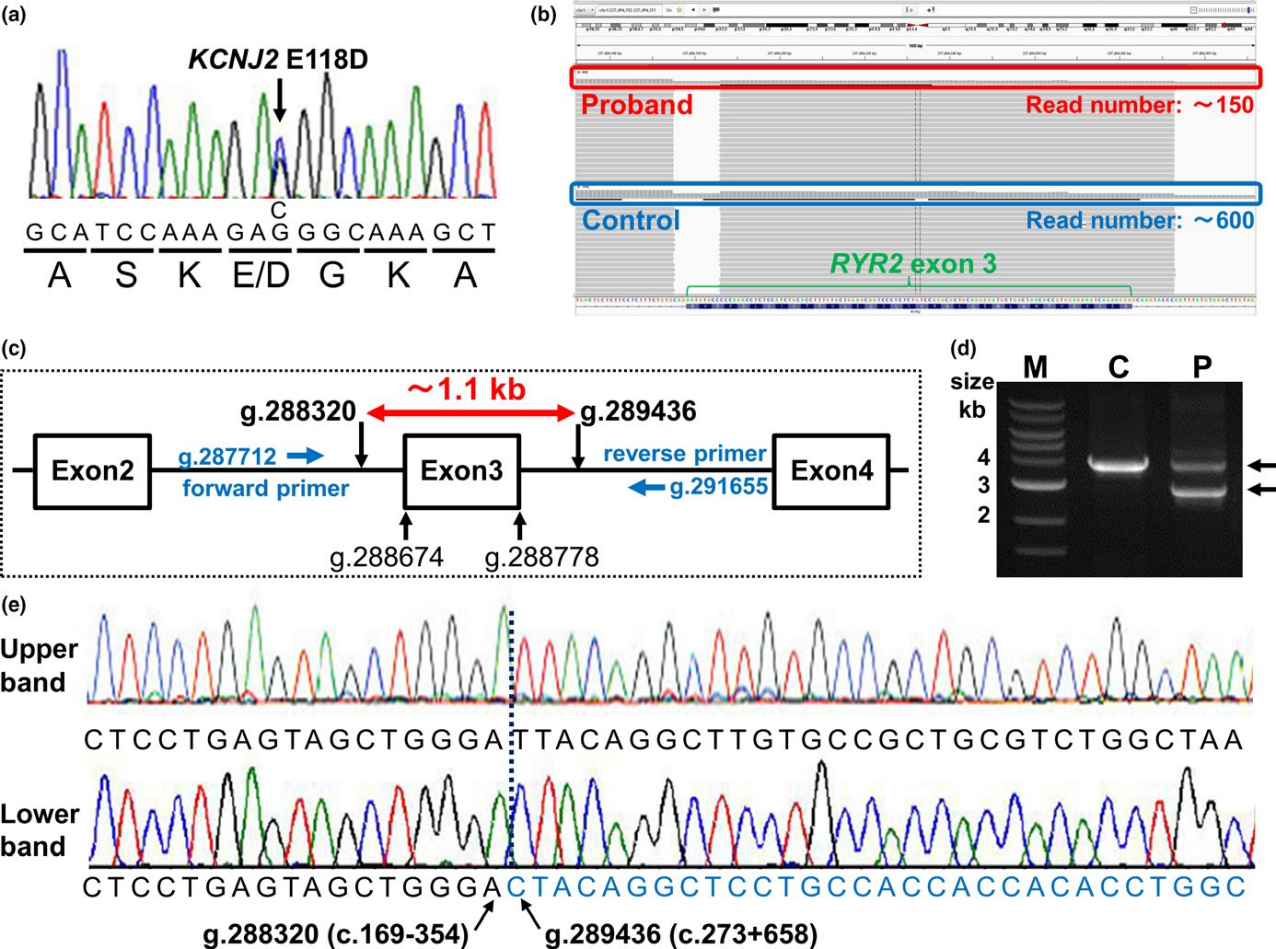
Identification of a *KCNJ2* E118D variant and a novel exon3 deletion of *RYR2*. (a) A sequence electropherogram of *KCNJ2* gene of the proband (Ⅲ‐3) (Figure 1). Nucleotide and amino acid substitutions were indicated below the electropherogram. (b) A result of targeted panel sequencing of the proband and control showing read number around exon3 of *RYR2*. Red and blue enclosing lines indicate read number of the proband and control, respectively. (c) Schematic representation of the position of exon3 of *RYR2* (NCBI Reference Sequence NC_000001.10), deletion sites, and primers used for long‐range PCR (d). (d). Electrophoresis of long‐range PCR products. M, marker; C, control; P, proband. (e) Direct sequencings of the upper band (upper panel) and lower band (lower panel) from the proband (d).

On the first genetic screening by the targeted panel sequencing of the proband (Ⅲ‐3), we identified only a rare *KCNJ2* (NM_000891.1) E118D variant (Figure 4a) (rs538725136, allele frequency in East Asian: 0.0004622 in Exome Aggregation Consortium Browser [http://exac.broadinstitute.org/] and that in Japanese: 0.0017 in Human Genetic Variation Database [http://www.hgvd.genome.med.kyoto-u.ac.jp/]) and failed to identify any other disease‐causing variants including *RYR2*.

Since the proband presented with typical CPVT phenotype, although *KCNJ2* variants can be the cause of an atypical type of CPVT,(Tester et al., 2006) we next proceeded to conduct pair analysis to detect the presence of CNVs.(Ohno et al., 2014) Read numbers obtained by the targeted panel sequencing were compared between control and the proband, then we found that the read number around exon3 in *RYR2* of the proband was apparently reduced compared to that of control (Figure 4b), suggested the presence of CNV, massive exon3 deletion, in the proband.

To confirm it, we conducted long‐range PCR using primers designed in intron 2 (forward primer) and intron 3 (reverse primer) of *RYR2* as shown in Methods and Figure 4c. Electrophoresis of the PCR products from the control showed single band which size was consistent with an expected one (approximately 3.9 kb) whereas those from the proband showed two distinct bands (Figure 4d). The size of the upper band from the proband was approximately 3.9 kb identical to that from the control, and the size of the lower band was approximately 2.8 kb (Figure 4d), suggesting the presence of ~1.1 kb deletion of *RYR2* including exon3. Direct sequencing of the upper band showed the sequence of wild‐type* RYR2* including exon3 (Figure 4e). On the other hand, that of the lower band displayed the site of the deletion of *RYR2* from intron2 to intron3 (Figure 4e). Thus, we finally identified a novel exon3 deletion of *RYR2*, NC_000001.10 (NM_001035.2):c.169‐353_273+657del (*RYR2*‐exon3 deletion) which was different from previous reports. (Bhuiyan et al., 2007; Marjamaa et al., 2009; Medeiros‐Domingo et al., 2009; Ohno et al., 2014; Szentpali, Szili‐Torok, & Caliskan, 2013).

As shown in the pedigree (Figure 1), her father (Ⅱ‐2) and paternal aunt (Ⅱ‐4) were also found to carry both the *RYR2*‐exon3 deletion and *KCNJ2* E118D. Her asymptomatic 11‐year‐old daughter (Ⅳ‐3) carried the *RYR2*‐exon3 deletion alone, but her sons (Ⅳ‐1 and Ⅳ‐2) carried neither the *RYR2*‐exon3 deletion nor the *KCNJ2* E118D.

### 3.3 Functional analysis of the *KCNJ2* E118D variant

**Figure 5:**
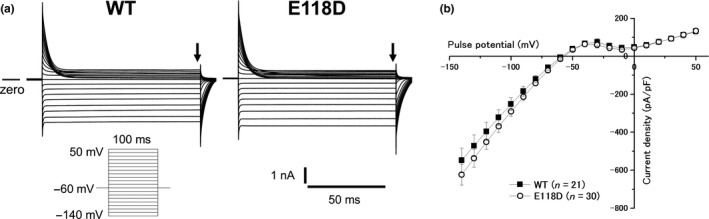
Electrophysiological properties for KCNJ2 WT and KCNJ2 E118D. (a) Representative currents obtained by a pulse protocol shown in the inset for KCNJ2 WT (left panel) and KCNJ2 E118D (right panel). (b) Current–voltage relationships for KCNJ2 WT (filled squares, *n* = 21) and KCNJ2 E118D (open circles, *n* = 30). Current amplitudes were evaluated at the end of the test pulses (arrows in A). WT, wild‐type

Since *KCNJ2* variants can be the cause of an atypical type of CPVT,(Tester et al., 2006) there remained a possibility that the *KCNJ2* E118D variant might cause the CPVT phenotype of this family. Therefore, we performed functional analysis of the variant. We expressed KCNJ2 WT or KCNJ2 E118D in COS‐1 cells and recorded whole‐cell currents (I_K1_). KCNJ2 E118D exhibited typical I_K1_ indistinguishable from KCNJ2 WT (Figure 5a). Inward rectification property and I_K1_ density for KCNJ2 E118D were almost the same as those for KCNJ2 WT (Figure 5b). I_K1_ density at −140 mV for WT and E118D was −430 ± 79 pA/pF (*n* = 21) and −484 ± 70 pA/pF (*n* = 30), respectively (*p* = 0.17).

**Figure 6:**
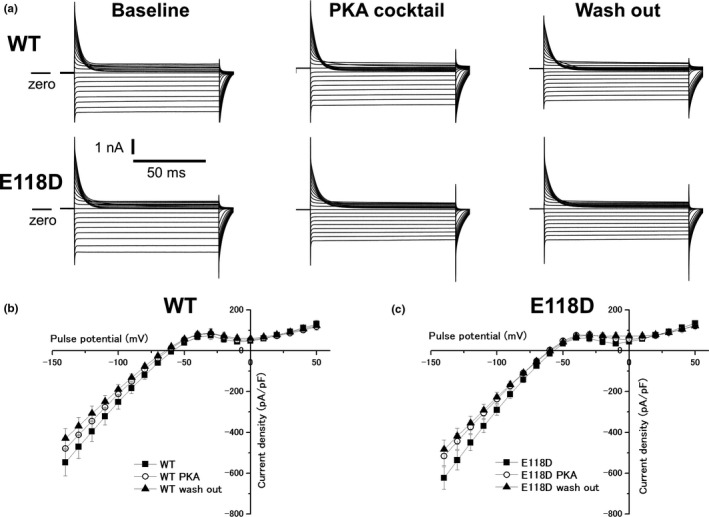
Effects of catecholaminergic stimulation on expressed currents (I_K1_) for KCNJ2 WT and KCNJ2 E118D. (a) Representative currents for KCNJ2 WT (upper panels) and KCNJ2 E118D (lower panels) obtained by the pulse protocol shown in the inset of Figure 5a. Currents were recorded at baseline (left panels), in the presence of a PKA cocktail (middle panels) and after wash out of it (right panels). (b) Current–voltage relationships for KCNJ2 WT obtained at baseline (filled squares, *n* = 21), in the presence of the PKA cocktail (open circles, *n* = 21) and after wash out of it (filled triangles, *n* = 21). (c) Current–voltage relationships for KCNJ2 E118D obtained at baseline (filled squares, *n* = 30), in the presence of the PKA cocktail (open circles, *n* = 30) and after wash out of it (filled triangles, *n* = 30). WT, wild‐type; PKA, protein kinase A

We next examined the effects of protein kinase A (PKA) activation on I_K1_ to resemble the catecholaminergic effect which occurs during exercise‐induced VAs. After recording I_K1_ at baseline condition, I_K1_ was also recorded in the presence of PKA cocktail (100 μmol/L forskolin +10 μmol/L 3‐isobutyl‐1‐methylxanthine) and after wash out of it, as reported by Vega et al.(Vega et al., 2009) As shown in Figure 6, PKA activation affected neither KCNJ2 WT nor KCNJ2 E118D. Contrary to the report by Vega et al.,(Vega et al., 2009) PKA activation did not reduce even I_K1_ produced by KCNJ2 WT.

## 4 DISCUSSION

### 4.1 Identification of a rare *KCNJ2* E118D variant and a novel exon3 deletion of *RYR2*

Our genetic screening by the initial targeted panel sequencing on the proband with CVPT identified only a rare *KCNJ2* E118D variant. However, focusing on the read numbers of the targeted panel sequencing led us think of the presence of the CNV, then we could finally identify a novel massive deletion of *RYR2* including exon3.

An MLPA method has been reported to be useful for identifying CNVs for many diseases including arrhythmogenic disorders. However, it is noteworthy that we identified CNV in *RYR2* by focusing on the read numbers of the targeted panel sequencing and subsequent long‐range PCR without performing the MLPA method. (Barc et al., 2011; Cox et al., 2011; Sonoda et al., 2017, 2018) Therefore, our study underlies the diagnostic importance of examining the read numbers in targeted panel sequencing to rule out the presence of pathological CNVs, even when a conventional method identifies candidate variants that are not actually disease‐causing.

Although there have been several reports regarding massive deletion of *RYR2* including exon3, direct sequencing of the PCR products (Figure 4e) from the proband revealed that the deletion site of our *RYR2*‐exon3 deletion was different from those of previous reports.(Bhuiyan et al., 2007; Marjamaa et al., 2009; Medeiros‐Domingo et al., 2009; Ohno et al., 2014; Szentpali et al., 2013).

### 4.2 Pathophysiological roles of U‐waves in patients with CPVT

In patients with *RYR2*‐related CPVT, intracellular Ca^2+^ overload or oscillations in the heart are thought to be induced by catecholaminergic stimulation through an increased diastolic Ca^2+^ leakage from SR, which may lead to DAD or DAD‐induced triggered activity.(Jiang et al., 2004; Katra & Laurita, 2005; Katra et al., 2007; Paavola et al., 2007) Clinical examinations of CPVT patients reported that DADs recorded in monophasic action potential of ventricular myocardium could be augmented by catecholaminergic stimulation, and concomitant U‐wave changes on ECG were associated with the DADs.(Nakajima et al., 1997; Paavola et al., 2007) In the present case, catecholaminergic stimulation by isoproterenol infusion augmented U‐waves, followed by PVC. These findings appear to be consistent with these concepts. Intriguingly, Aizawa et al. reported that U‐waves in CPVT patients could also be affected by some specific conditions such as after ventricular pacing, after the exercise test and after sinus arrest.(Aizawa et al., 2006) Therefore, there may be a high probability of the relationship between U‐waves and arrhythmogenicity in CPVT patients; however, it needs to be further investigated.

### 4.3 Phenotypic manifestations of this family

The majority of *RYR2* variants identified in typical CPVT patients are missense mutations. However, carriers of exon3 deletions of *RYR2* have been reported to show a distinct phenotype of CPVT characterized by concomitant SND, AVB, atrial fibrillation, atrial standstill, dilated cardiomyopathy, and LVNC/hypertrabeculation.(Bhuiyan et al., 2007; Marjamaa et al., 2009; Medeiros‐Domingo et al., 2009; Ohno et al., 2014; Szentpali et al., 2013).

In this family, the proband and her elder sister experienced cardiac events during exercise. The proband had sinus bradycardia, prominent U‐waves at rest, and hypertrabeculation localized at LVA. Her father and paternal aunt received PPM due to SND, and had prominent U‐waves at rest. Her father also had AT with AVB. These family members, except for her deceased sister, carried both the *RYR2*‐exon3 deletion and the *KCNJ2* E118D, which obscured pathophysiological roles of each variant. On the other hand, ECG of her daughter (age 11), who carried only the *RYR2*‐exon3 deletion, showed no prominent U‐waves but inverted T‐waves. We speculate that normal juvenile pattern of T‐waves might obscure U‐waves.(Surawicz, 1998) She had been asymptomatic, and exercise stress test induced only one premature ventricular contraction. Intriguingly, she also had hypertrabeculation at LVA. These various phenotypic manifestations, except for prominent U‐waves at rest, of this family appeared to be consistent with those with exon3 deletion of *RYR2* in previous reports.(Bhuiyan et al., 2007; Marjamaa et al., 2009; Medeiros‐Domingo et al., 2009; Ohno et al., 2014; Szentpali et al., 2013).

### 4.4 Possible functional role of the *RYR2*‐exon3 deletion and the *KCNJ2* E118D variant

The *RYR2*‐exon3 deletion may produce mutant RyR2 proteins without frameshift, thus may cause their functional abnormalities. Actually, functional analysis of exon3 deletion of RyR2 using heterologous expression in HEK293 cells or HL‐1 cells revealed that it reduced the endoplasmic reticulum or SR luminal Ca^2+^ threshold at which Ca^2+^ release terminates and increased the fractional Ca^2+^ release, and also enhanced the amplitude of store overload‐induced Ca^2+^ transients.(Tang, Tian, Wang, Fill, & Chen, 2012) Considering it together with clinical reports of patients with exon3 deletion of *RYR2*, various phenotypic manifestations in this family could be attributable to the *RYR2*‐exon3 deletion.(Bhuiyan et al., 2007; Marjamaa et al., 2009; Medeiros‐Domingo et al., 2009; Ohno et al., 2014; Szentpali et al., 2013).

Regarding the *KCNJ2* E118D variant, it caused no functional abnormalities of I_K1_ based on the patch‐clamp study, even in the presence of PKA activation. These findings suggested that prominent U‐waves at rest in this family might be associated with the *RYR2*‐exon3 deletion.

## 5 CONCLUSIONS

A novel *RYR2*‐exon3 deletion and a rare *KCNJ2* E118D variant were identified in a family with CPVT. The *RYR2*‐exon3 deletion could be identified by focusing on the read numbers of the targeted panel sequencing without performing the MLPA method, which emphasizes the importance of focusing on the read numbers when performing genetic analysis using NGS. The *RYR2*‐exon3 deletion may produce mutant RyR2 proteins without frameshift, thus cause RyR2 dysfunction, but the *KCNJ2* E118D variant did not cause I_K1_ dysfunction. Therefore, the novel *RYR2*‐exon3 deletion may be associated with phenotypic features of this family.

## CONFLICT OF INTEREST

The authors declare no conflict of interest.
